# Phenotyping patterns of left ventricular remodeling and hypertrophy in systemic hypertension by cardiac magnetic resonance (CMR)

**DOI:** 10.1186/1532-429X-17-S1-P323

**Published:** 2015-02-03

**Authors:** Jonathan C Rodrigues, Stephen Lyen, Amardeep Ghosh Dastidar, Neelam Hassan, Amy E Burchell, Laura E Ratcliffe, Emma C Hart, Chiara Bucciarelli-Ducci, Mark Hamilton, Julian F Paton, Angus K Nightingale, Nathan E Manghat

**Affiliations:** 1 CMR Unit, NIHR Cardiovascular Biomedical Research Unit, Bristol Heart Institute, Bristol, UK; 2 School of Physiology and Pharmacology, The University of Bristol, Bristol, UK; 3 Foundation School, Severn Postgraduate Deanery, Bristol, UK; 4 Cardionomics Research Group, Bristol Heart Institute, Bristol, UK

## Background

CMR is the non-invasive, gold standard for quantification of left ventricular mass (LVM) and ventricular volumes. We sought to investigate the patterns of remodeling and hypertrophy in patients with systemic hypertension using CMR.

## Methods

Consecutive patients referred from our tertiary hypertension clinic, who underwent CMR at 1.5T, were included. Exclusion criteria included patients with clinical or CMR evidence of concomitant pathology (e.g. moderate-severe aortic stenosis) which may confound remodeling/hypertrophy pattern. Indexed LVM (iLVM), including papillary muscle mass by blood pool thresholding, indexed LV end-diastolic volume (iEDV) and ejection fraction (EF) were calculated using established CMR methods and normalized to body surface area. Values out-with the 95^th^ confidence intervals of established CMR normal reference values were considered abnormal. Mass : volume ratio (M/V) >1.12 for men and >1.14 for women was defined as abnormal, in accordance with previous literature. The phenotypes of ventricular remodeling and hypertrophy were defined as either normal, concentric remodeling, asymmetric remodeling, concentric hypertrophy, asymmetric hypertrophy, eccentric hypertrophy or decompensation depending on the constellation of iLVM, iEDV, M/V, asymmetric thickness (>13mm and >1.5 fold opposing wall) and EF

## Results

**Figure 1:**
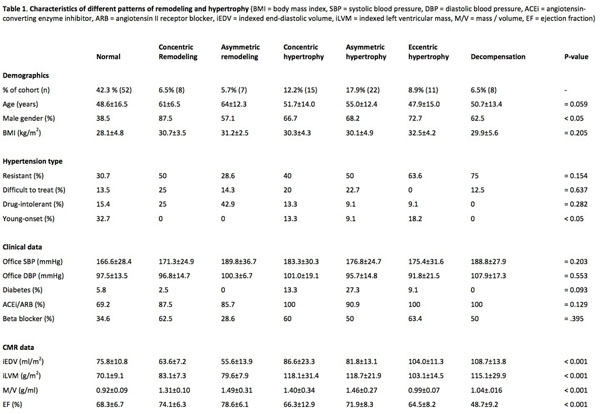
Characteristics of different patterns of remodeling and hypertrophy

One hundred and twenty three (n=123) patients were analysed. The prevalence of different phenotypical responses were as follows: normal (42.3%), concentric remodeling (6.5%), asymmetric remodeling (5.7%), concentric hypertrophy (12.2%), asymmetric hypertrophy (17.9%) eccentric hypertrophy (8.9%) and decompensation (6.5%). The demographic and CMR characteristics of the different types of remodeling and hypertrophy are described in Figure 1. There was no predilection of remodeling/hypertrophic pattern according to hypertension type. 12.2% of our cohort had normal iLVM but demonstrated concentric/asymmetric remodeling. Subgroup analysis by remodeling (n=15) versus hypertrophy (n=22) revealed no significant difference in age (62±9.4 vs 55.1±12.4 years, p=0.0598), gender (% male 74.4% vs 68.2%, p=0.999), BMI (30.9±3.0 vs 30.1±4.9 kg/m^2^, p=0.5836), degree of hypertension (SBP 179.9±31.3 vs 176.8±24.7 mmHg, p=0.7407 and DBP 98.4±11.4 vs 95.7±14.8 mmHg, p=0.5557) or prevalence of potentially remodeling modifying medication (ACEi/ARB 80.0% vs 77.3%, p=0.999).

## Conclusions

Varied CMR patterns of LV remodeling/hypertrophy occur in hypertensive patients with no predilection demonstrated in subgroup analysis. CMR-derived iLVM is increasingly used an end-point for clinical trials in hypertension. Our data suggest that patterns of LV remodeling/hypertrophy should also be taken into account to avoid misclassifying patients with normal iLVM (but abnormal ventricles due to remodeling) together with patients with normal iLVM and truly normal ventricles.

## Funding

NIHR Cardiovascular Biomedical Research Unit, Bristol Heart Institute

JCLR: Clinical Society of Bath Postgraduate Research Bursary

ECH: BHF grant IBSRF FS/11/1/28400.

